# Evaluation of cystatin C as an early biomarker of cadmium nephrotoxicity in the rat

**DOI:** 10.1007/s10534-015-9903-3

**Published:** 2015-12-29

**Authors:** Walter C. Prozialeck, Aaron VanDreel, Christopher D. Ackerman, Ian Stock, Alexander Papaeliou, Christian Yasmine, Kristen Wilson, Peter C. Lamar, Victoria L. Sears, Joshua Z. Gasiorowski, Karyn M. DiNovo, Vishal S. Vaidya, Joshua R. Edwards

**Affiliations:** Department of Pharmacology, Midwestern University, 555 31st Street, Downers Grove, IL 60515 USA; Department of Biomedical Sciences, Midwestern University, 555 31st Street, Downers Grove, IL 60515 USA; Department of Physiology, Midwestern University, 555 31st Street, Downers Grove, IL 60515 USA; Renal Division, Brigham and Woman’s Hospital, Harvard Medical School, Boston, MA 02115 USA

**Keywords:** Cadmium, Biomarkers, Cystatin C, Nephrotoxicity, Proximal tubule

## Abstract

Cadmium (Cd) is a nephrotoxic environmental pollutant that causes insidious injury to the proximal tubule that results in severe polyuria and proteinuria. Cystatin C is a low molecular weight protein that is being evaluated as a serum and urinary biomarker for various types of ischemic and nephrotoxic renal injury. The objective of the present study was to determine if cystatin C might be a useful early biomarker of Cd nephrotoxicity. Male Sprague–Dawley rats were given daily injections of Cd for up to 12 weeks. At 3, 6, 9 and 12 weeks, urine samples were analyzed for cystatin C, protein, creatinine, β_2_ microglobulin and kidney injury molecule-1. The results showed that Cd caused a significant increase in the urinary excretion of cystatin C that occurred 3–4 weeks before the onset of polyuria and proteinuria. Serum levels of cystatin C were not altered by Cd. Immunolabeling studies showed that Cd caused the relocalization of cystatin C from the cytoplasm to the apical surface of the epithelial cells of the proximal tubule. The Cd-induced changes in cystatin C labelling paralleled those of the brush border transport protein, megalin, which has been implicated as a mediator of cystatin C uptake in the proximal tubule. These results indicate that Cd increases the urinary excretion of cystatin C, and they suggest that this effect may involve disruption of megalin-mediated uptake of cystatin C by epithelial cells of the proximal tubule.

## Introduction

Cd is an important industrial and environmental pollutant that can damage various organs including the lung, liver, kidney and bone (ATSDR 2008; Jarup and Akesson 2009). In addition, Cd can act as an endocrine disruptor and it is carcinogenic (ATSDR 2008; Byrne et al. 2009; Joseph 2009). With the chronic, low-level patterns of exposure that are common in humans, the kidney is the primary target of toxicity, where Cd accumulates in the epithelial cells of the proximal tubule, resulting in a generalized reabsorptive dysfunction characterized by polyuria and low molecular weight proteinuria (Jarup 2002; Jarup and Akesson 2009; Prozialeck and Edwards 2010, 2012). In addition, some evidence suggests that Cd-induced kidney injury can result in alterations (either increases or decreases) in glomerular function (Weaver et al. 2011). These effects can result from even relatively low levels of exposure, and children and individuals with confounding health conditions, such as diabetes, may be especially vulnerable (Akesson et al. 2005; ATSDR 2008; de Burbure et al. 2006; Edwards and Prozialeck 2009; Friedman et al. 2006; Navas-Acien et al. 2009; Trzeciakowski et al. 2014).

As a result of its ability to accumulate Cd and its sensitivity to injury, the kidney is, in effect, a sentinel of Cd exposure, and much attention has been focused on the identification of the early stages of Cd nephrotoxicity. However, this issue has been complicated by the fact that many standard metrics for identifying kidney injury such as blood urea nitrogen (BUN), serum creatinine and urinary protein are not sensitive markers of Cd-induced kidney injury. Over the years, several other urinary biomarkers have been found to be more sensitive indicators of Cd-nephrotoxicity. Some of the biomarkers that have proven to be most useful include low molecular weight proteins such as β_2_-microglobulin and metallothionein, and the proximal tubule-derived enzyme *N*-acetyl-β-d-glucosaminidase (NAG) (For reviews see, Jin et al. 1999; Kawada et al. 1995; Moriguchi et al. 2009; Prozialeck 2013; Prozialeck and Edwards 2010, 2012). However, even though these markers have been used to monitor Cd toxicity in humans and experimental animals, several problems remain. For example, these markers only identify relatively late stages of Cd-induced injury. By the time changes in these markers become evident, the effects of Cd on the kidney may be severe and irreversible (Prozialeck and Edwards 2012; Wu et al. 2011). Moreover, each of these agents possesses some unusual properties that complicate their use as markers of Cd nephrotoxicity. For example, the urinary excretion of metallothionein reflects both Cd exposure as well as proximal tubular injury; identifying the critical level of urinary metallothionein to indicate the onset of tubular injury has been problematic (Prozialeck 2013; Prozialeck and Edwards 2010). With regard to β_2_-microglobulin, it is not totally clear if increases in its urinary excretion are solely the result of proximal tubule dysfunction or may also reflect plasma levels of the protein, which can be influenced by actions of Cd at the glomerulus or on organs other than the kidney. A major problem with NAG is that enzyme activity can be affected by other substances, particularly toxic metals such as Hg, that might also be present in urine of metal-exposed subjects (Prozialeck 2013; Prozialeck and Edwards 2010). As a result of these problems, there is a need for more sensitive markers of Cd nephrotoxicity. One molecule that shows significant potential in this context is cystatin C.

Cystatin C is a 13 kDa cysteine protease inhibitor that is produced by cells throughout the body. Cystatin C is abundant in serum/plasma and is readily filtered at the glomerulus. Serum levels of cystatin C have been proposed as a useful marker to estimate glomerular filtration rate (Filler et al. 2005; Ghys et al. 2014; Parikh et al. 2010; Parikh and Devarajan 2008; Peralta et al. 2011; Vaidya et al. 2008). Unlike creatinine, cystatin C is not secreted by the proximal tubule. Instead, filtered cystatin C is taken up through a megalin-dependent process and then completely catabolized in proximal tubule epithelial cells (Ghys et al. 2014). Serum levels of cystatin C are much less likely to be affected by factors such as gender, metabolic status or disease states than are levels of creatinine (Ghys et al. 2014; Ozer et al. 2010; Parikh et al. 2010, 2011; Parikh and Devarajan 2008; Vaidya et al. 2008).

Changes in both serum and urinary levels of cystatin C have been observed in various models of kidney injury (Dieterle et al. 2010; Ghys et al. 2014; Parikh et al. 2010; Vaidya et al. 2008; Zhang et al. 2011). Several equations for estimating glomerular filtration based on serum levels of cystatin C have been developed, and there is evidence that these cystatin C-based measurements may out-perform traditional creatinine-based estimates of GFR (Ghys et al. 2014; Inker and Okparavero 2011; Stevens et al. 2010; Weinert et al. 2011; Zhang et al. 2011). However, there is also some evidence that proteinuria and proximal tubular damage may influence the urinary excretion of cystatin C and affect cystatin C-based estimates of renal function (Dieterle et al. 2010; Kim et al. 2012).

Cystatin C has recently received some attention as a potential biomarker of Cd nephrotoxicity (Harisa et al. 2014; Poreba et al. 2011; Shelley et al. 2012; Wallin et al. 2014; Weaver et al. 2011, 2014; Wu et al. 2011). However, those studies focused primarily on the measurement of serum levels of cystatin C as an indicator of glomerular function and they yielded equivocal and somewhat conflicting results. Most of the studies did not include data on the possible effects of Cd on the urinary excretion of cystatin C. In our own preliminary studies utilizing a subchronic model of Cd exposure in rats, we found significant increases in urinary cystatin-C that occurred with even relatively short-term Cd exposure (VanDreel et al. 2012). In addition, Immunolabeling studies revealed significant alterations in the patterns of cystatin-C localization in the proximal tubule epithelium. In light of these preliminary findings, additional studies on the effects of Cd on serum and urinary levels of cystatin C seemed warranted.

The present studies were aimed at further characterizing the possible utility of cystatin C as an early urinary and/or serum biomarker of Cd nephrotoxicity. In addition, we examined the effects of Cd on the localization of cystatin C in the kidney. These studies involved the use of a well-established model of long-term Cd-exposure in the rat and the direct comparison of the cystatin C results with those of a panel of standard markers of Cd toxicity and kidney function (β_2_ microglobulin, Kim-1, total protein and creatinine).

## Materials and methods

Animal work was conducted in compliance with the United States NIH Guide for the Care and Use of Laboratory Animals (National Research Council of the National Academies 2011), and the studies were approved by the Institutional Animal Care and Use Committee of Midwestern University. Adult male Sprague–Dawley rats weighing 250–300 g (Harlan, Indianapolis, IN) were housed socially (two rats per plastic cage) on a 12 h/12 h light/dark cycle. For the low-dose, chronic studies, animals in the Cd treatment group (n = 6) received daily (Monday–Friday) subcutaneous injections of CdCl_2_ at a Cd dose of 0.6 mg (5.36 µmol)/kg in 0.25–0.35 ml isotonic saline for up to 12 weeks. Control group animals (n = 6) received daily injections of the saline vehicle alone. After 3, 6, 9 and 12 weeks of treatment, the animals were placed in individual metabolic cages and 24 h urine samples were collected. The animals were allowed free access to water at all times. Food was also available ad libitum, except during the period in which the urine samples were being collected. The 12 week Cd treatment protocol was repeated twice and data from the two treatment protocols were pooled whenever possible. In another study, groups of animals were treated with varying doses of Cd (0, 0.6 or 1.2, or 2.4 mg/kg) 5 days per week for 1–3 weeks. Before the start of each experiment, the Cd concentrations of all stock solutions used for the various treatment protocols were verified by Chemical Solutions, Inc. using the technique of inductively coupled plasma mass spectroscopy.

### Urine and blood analysis

After collection, the 24-h urine samples were aliquoted into 0.5–1.0 ml portions. The aliquots were frozen at −80 °C and later assayed for protein, creatinine, and the biomarkers of interest. In some cases, prior to freezing, the urine aliquots were stabilized in proprietary buffers and other reagents that are recommended for MAGPIX-based assays that were used for some of the analyses. Blood samples were obtained at the time the animals were euthanized and serum levels of cystatin C was determined by an enzyme-linked immunoabsorbent assay (ELISA) (R+D Systems) according to the manufacturer’s protocol.

### Biomarker determination

The urinary levels of cystatin C, Kim-1 and β_2_-microglobulin, were determined by microsphere-based Luminex x MAP technology using the MagPix xPONENT 4.1 equipment. The Multiplex technology allows for the determination of multiple analyses in a single sample and provides much greater sensitivities and dynamic ranges than commonly-used ELISA’s. This technique is similar to the assay that has been used to determine urinary levels of Kim-1 in our previous studies (Prozialeck et al. 2007, 2009a, b). Urinary levels of creatinine were determined by the colorimetric method of Shoucri and Pouliot (1977). With the dosing protocol used in these studies, Cd-treated animals tended to gain less weight than control animals (Prozialeck et al. 2007). Accordingly, all urinary parameters were expressed as units excreted per kg body weight per 24 h.

### Visualization of cystatin C and megalin

In our initial studies, cystatin C and megalin in individual tissue sections were visualized by separate immunohistochemical labeling procedures. Formalin-fixed, paraffin-embedded tissue sections (5 µm) were deparaffinized in xylene, rehydrated with a series of alcohol washes, and then incubated for 20 min at 95 °C in a citrate buffer solution (Vector Laboratories, #H3300). The sections were quenched for endogenous peroxidase by incubation in 0.3 % hydrogen peroxide in methanol at −20 °C. After quenching, the sections were blocked at room temp for 15 min in undiluted Background Reducing Buffer (Abcam, # ab64234). The samples were then incubated overnight at 4 °C in the primary antibodies, either a 1:100 dilution of goat anti-cystatin C (Abcam, # ab117642) or a 1:100 dilution of rabbit anti-megalin (Abcam, cat # ab76969).The primary antibodies were visualized using the avidin–biotin horseradish peroxidase technique according to the manufacturer’s protocol (Vector Laboratories, Vectastain Elite ABC Kit PK 6104 or PK6102). The samples were then counterstained with hematoxylin. Negative controls consisted of tissue sections that were incubated without the primary antibody.

In other experiments, a dual-labeling indirect confocal immunofluorescence procedure was used to visualize cystatin C and megalin in the same tissue sections. Fresh cryosections of rat kidney (5–20 µm) were fixed and permeabilized for 10 min in −20 °C methanol and then incubated for 15 min in undiluted Background Reducing Buffer (Abcam, # ab64234). The samples were then incubated overnight at 4 °C in the primary antibodies at a 1:100 dilution of goat anti-cystatin C (Abcam, # ab117642) or a 1:100 dilution of rabbit anti-megalin (Abcam, cat # ab76969). The samples were washed in PBS and incubated for 40 min in the secondary antibodies at a 1:200 dilution of TRITC conjugated chicken anti-rabbit IgG (Abcam, # ab6826) and a 1:100 dilution of Alexa Fluor488-conjugated donkey anti-goat IgG (Abcam, # ab150129). Samples were then washed in deionized water, mounted and viewed. Controls consisted of samples that were incubated without the primary antibodies.

For most of the studies, samples were viewed with a Nikon Eclipse E-400 microscope using a ×40 objective. Images were captured with a digital camera (Media Cybernetics) using automated exposure times and gain settings. The digital images were processed using the Image-Pro Plus image analysis software package (Media Cybernetics). To further characterize the patterns of cystatin C and megalin labeling in specific nephron segments, some of the samples were also examined using a Nikon Eclipse Ti microscope fitted with a Nikon A1R confocal interface with Nikon Elements C software on a HPZ400 computer. A stack of up to 100 images per section was acquired in 0.05 micron increments under a ×60 oil objective and representative images were selected from each stack.

Negative controls for all labeling studies consisted of kidney sections that were incubated without the primary antibodies, as well as sections that were incubated with non-diluted, non-immune serum from the same species in which the primary antibodies had been generated. To rule out the possibility that any apparent labeling may have resulted from fluorescence “spill over” when the FITC labeled samples were viewed with the TRITC filter configuration and vice versa, all experiments also included a group of samples that were labeled with each of the TRITC- or FITC-tagged antibodies individually. When the TRITC-labeled samples were viewed with the FITC filter configuration and the FITC-labeled samples were viewed with the TRITC filters, no labeling was evident in any of the samples. All labeling experiments were repeated at least three times and appeared to be highly reproducible.

### Image analyses

For the immunohistochemistry images in Fig. 5, the Image J computer program was used to create hand drawn regions of interest around each proximal tubule using the hematoxylin staining as a morphological and structural guide. Thresholds were set at (X-115) to identify pixels that were brown and above average background noise due to positively stained peroxidase signals from cystatin C and megalin. Total brown pixels for cross sections of each tubule were measured. A second region of interest was hand drawn within each tubule to capture 1–2 cells at the border of the cytoplasm and the cystatin C and megalin pixels above background. The percentages of apical/luminal cystatin C and megalin pixels were then calculated from the total pixels/tubule.

Fluorescence co-localization of cystatin C and megalin images in Fig. 6 was also measured using the Image J program. Megalin (red) and cystatin C (green) images were split into their separate channels. The average backgrounds were used to set thresholds for the red (25–255) and green (30–255) channels. The Coloc 2 plug-in was then used to determine the Pearson’s correlation and Manders split coefficients of control and Cd treated samples (online reference http://onlinelibrary.wiley.com/doi/10.1111/j.1365.tb03313.x/abstract).

### Statistical analysis

Statistical analyses were done using the GraphPad Prism Computer Program. Data for the various serum and urinary parameters were evaluated by two different types of analyses. The first approach involved the use of standard two-way ANOVA, followed by post hoc Tukey’s tests to determine the differences among the various treatment groups, whenever the p value for the ANOVA was less than 0.05. This was done because similar tests have been commonly used in many of the previous studies on the urinary excretion of these markers in similar animal models (Arsalane et al. 1999; Bernard and Hermans 1997; Lee et al. 1983; Lermioglu and Bernard 1998; Sugihira et al. 1986). However, while performing these analyses, we found that this approach might not be appropriate because the data points for the later treatment periods showed significantly more variability than the points for the earlier treatment periods. ANOVA and Tukey’s tests are parametric tests that assume normality of distribution and homogeneity of variances (i.e. that SD’s are equal for all treatment groups) (Zar 1996). Analysis of the present data by the Kolmogorov–Smirnov test (normality) and Levene’s test (homogeneity of variances) showed that much of the data did not meet these criteria. Accordingly, the data were also analyzed by the non-parametric Kruskal–Wallis test and Dunn’s post hoc test for multiple comparisons, which are more appropriate for these types of variable, heterogeneous data (Zar 1996). Since many previous studies on these markers utilized parametric methods, we have included the results of both our parametric and non-parametric analysis so that the reader can directly compare the results of this study with those previously reported in the literature.

## Results

**Fig. 1:**
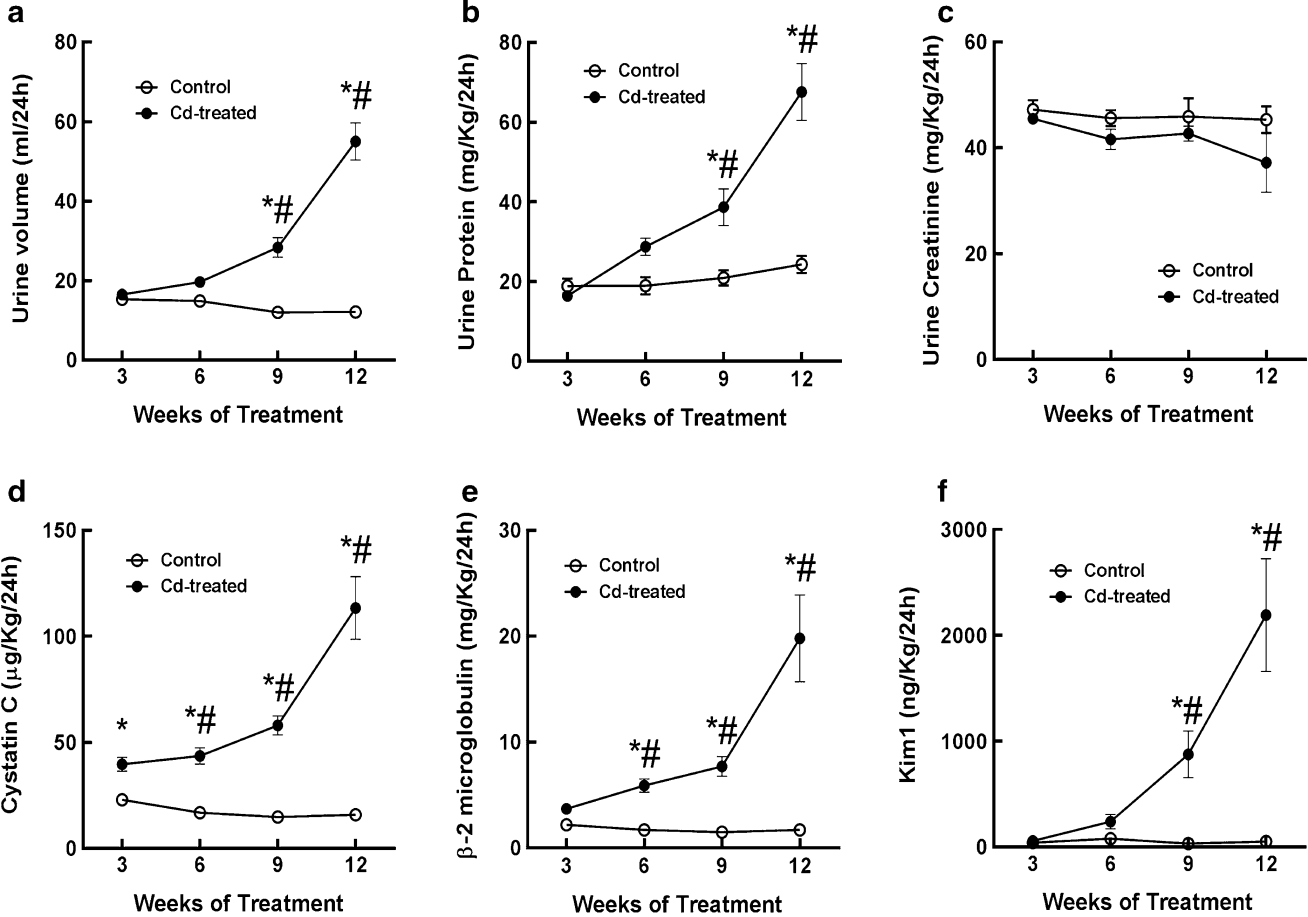
Effects of Cd on urine volume, urinary creatinine, protein, cystatin C, β_2_ microglobulin and Kim-1. Male Sprague–Dawley rats received daily subcutaneous injections of Cd (0.6 mg/kg) for up to 12 weeks, and 1 day at weeks 3, 6, 9 and 12, 24 h urine samples were collected and analyzed for creatinine, protein and the various biomarkers as described under “Materials and methods” section. An *asterisk* indicates significant differences from week matched control values as determined by 2-way ANOVA (p < 0.05) and Tukey’s post hoc test. A # indicates significant differences from week matched control values as determined by the non-parametric Kruskal–Wallis test (p < 0.05) and Dunn’s test for multiple comparisons. Values represent the mean ± SEM; n = 11–24 for each data point

Figure 1 shows the effects of Cd (0.6 mg/kg, 5 days per week) on urine volume (a) and the urinary excretion of protein (b), creatinine (c) and a panel of biomarkers including cystatin C (d), β_2_ microglobulin (e) and Kim-1 (f). As may be seen, treatment with Cd, increased urine volume (graph a) and total urinary protein excretion (graph b). These changes reached levels of statistical significance after about 9 weeks of Cd exposure. Cd had no significant effect on the urinary excretion of creatinine even after 12 weeks (graph c). The development of polyuria and proteinuria, with no change in creatinine excretion is characteristic of Cd-induced proximal tubule dysfunction (Prozialeck et al. 2007). The urinary excretion of cystatin C was significantly elevated at 3–6 weeks, depending on the statistical method that was used and continued to increase throughout the remainder of the 12 week treatment protocol (graph d). With the parametric statistical tests (2 way-ANOVA and Tukey’s post hoc test), urinary cystatin C levels were significantly elevated after only 3 weeks. However, as noted in “Materials and methods” section, such parametric tests are not totally appropriate for this type of data where variances are not homogeneous. When the more appropriate non-parametric Kruskal–Wallis and Dunn’s post hoc tests were used, the levels of cystatin C were significantly elevated after 6 weeks of exposure. The time course for the increase in cystatin C excretion was similar to that of the classic Cd biomarkers β_2_ microglobulin (graph e) and the more-recently described marker Kim-1 (graph f). It should be noted that in the present study, even though Kim-1 appeared to be slightly increased after as little as 6 weeks into the treatment protocol, the increase did not reach a level of statistical significance until 9 weeks. Our previous studies with Kim-1 using this same model showed significant increases in urinary Kim-1 after only 6 weeks. We cannot explain the reason for this slight discrepancy. However, it is interesting to note that when the Kim-1 data from weeks 3 and 6 of the present study were analyzed without inclusion of the 9 and 12 week data, the increase in Kim-1 was statistically significant (p < 0.05) at 6 weeks. This illustrates the fact that as the variance of the biomarker data increases with longer exposure times, the statistical significance of changes that might be evident at earlier time points can be obscured. In this context, the significance of results of a particular biomarker at any given time point is to a certain extent dependent on the duration of exposure in the overall study.

Nevertheless, the salient point from this data as a whole is that cystatin C appears to be a very early urinary marker of the Cd toxicity. It should also be noted that our observed values for cystatin C, Kim-1 and β_2_ microglobulin were also within reference ranges provided by the manufacturer of the reagents (Millipore Inc.). Moreover, similar values for urinary cystatin C, β_2_ microglobulin and Kim-1 have been reported by other investigators (Cardenas-Gonzalez et al. 2013; Dieterle et al. 2010; Gautier et al. 2014a, b; Ghys et al. 2014; John-Baptiste et al. 2012; Spanu et al. 2014; Zhou et al. 2008).

The results of the foregoing analyses showed that Cd caused significant increases in the urinary excretion of cystatin C as early as 3–6 weeks into the treatment period, which is 3–6 weeks before the onset of overt signs of Cd nephrotoxicity such as polyuria and proteinuria. The increased excretion of cystatin C occurred in the same general time frame as the increase in urinary excretion of β_2_ microglobulin and Kim-1 which have both been used as sensitive urinary markers of Cd nephrotoxicity (Prozialeck et al. 2007; Prozialeck 2013; Prozialeck and Edwards 2010; Ruangyuttikarn et al. 2013).

**Fig. 2:**
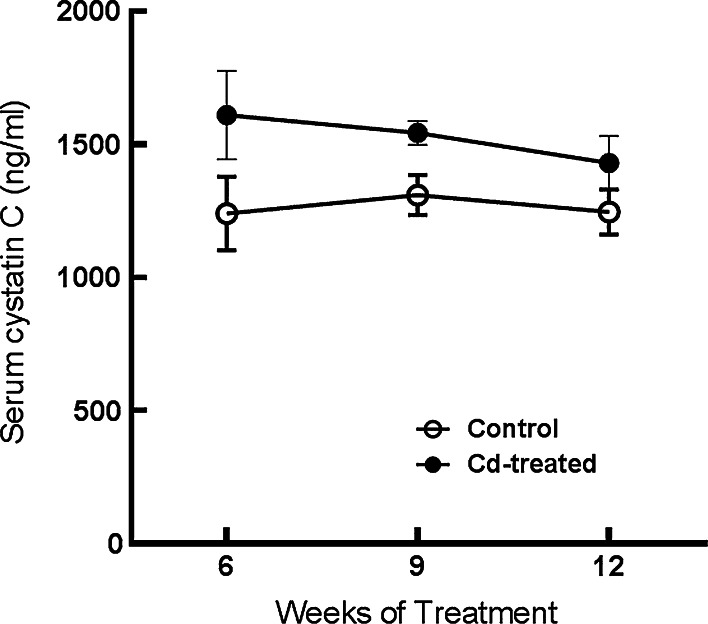
Lack of effects of Cd on serum cystatin C. Animals received daily subcutaneous injections of Cd (0.6 mg/kg) as described in “Materials and methods” section. Animals were euthanized at 6, 9 and 12 weeks and serum was collected and analyzed for cystatin C as described in “Materials and methods” section. The values represent the mean ± SEM; n = 6–11 for each data point

As may be seen in Fig. 2, Cd had no effect on serum levels of cystatin C, which are thought to reflect glomerular filtration rate. As we have shown in our previous studies using this same treatment protocol, Cd also has no effect on serum levels of creatinine, which is a classical marker of glomerular function (Prozialeck et al. 2007, 2009a, b).

**Fig. 3:**
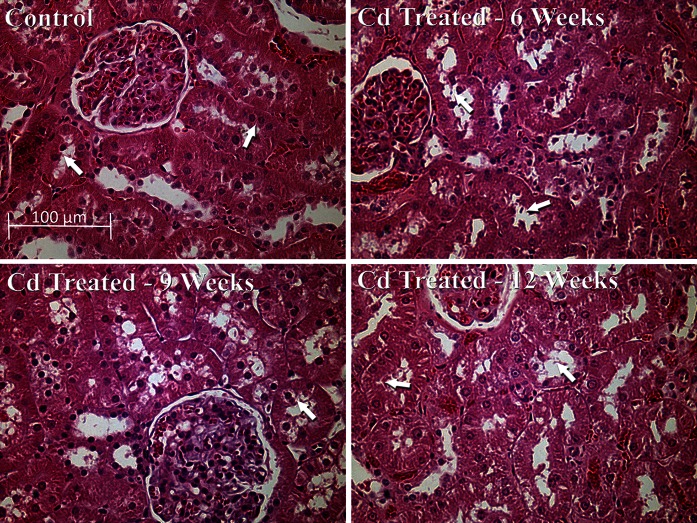
Effects of Cd on general morphology of the renal cortex. Rats were treated with Cd (0.6 mg/kg/day) for 6, 9 or 12 weeks as described in “Materials and methods” section and representative 5 µm thick sections of the renal cortex processed for H+E staining. The *scale bar in the top right figure* represents 100 µm

Figure 3 shows hematoxylin and eosin-stained tissue sections of outer renal cortex from control and Cd-treated animals. Note that proximal tubule epithelial cells in the control samples (top left) exhibited cuboidal shapes, well-defined nuclei, and a uniform cytoplasm, with no spaces or gaps between the cells (see arrows). By contrast, the epithelial cells in the samples from Cd-treated animals (top right and bottom row) showed a ragged, irregular appearance, with gaps between the cells (see arrows). The cells remained attached to the basement membrane, however, and showed little overt evidence of necrosis. Analyses of the glomeruli and distal segments of the nephron revealed no evidence of pathology. Together, these observations along with the urinary and serological results described above are consistent with previous findings that in this particular animal model Cd primarily damages the proximal tubule while having little effect on glomerular structure or function (Prozialeck et al. 2007, 2009a, b).

**Fig. 4:**
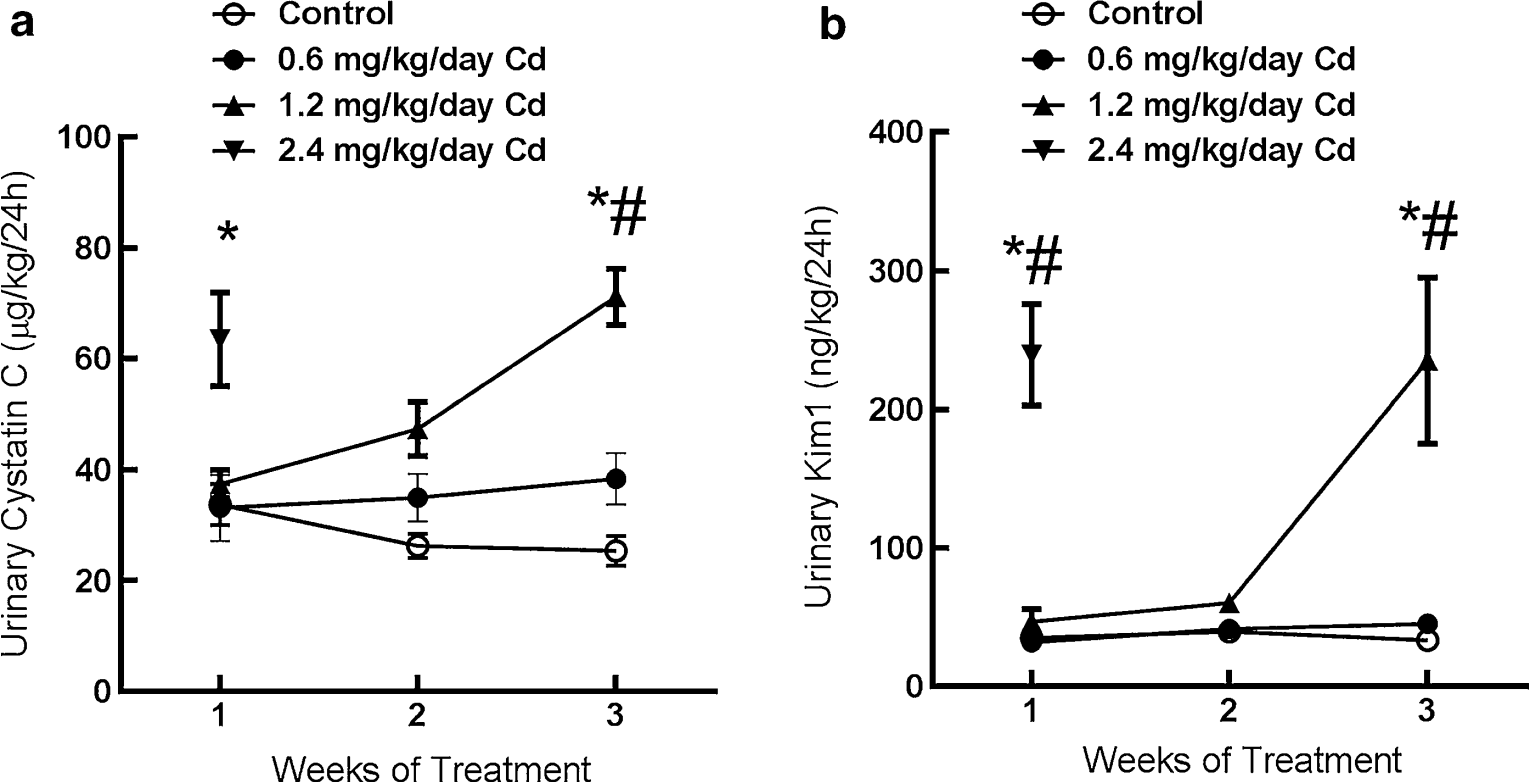
Dose-dependence of the Cd-induced increase in cystatin C and Kim-1 excretion. Animals were treated with varying doses of Cd (0, 0.6, 1.2 or 2.4 mg/kg/5 days per week for 4 weeks) and urine was analyzed for levels of cystatin C and Kim-1 as described in “Materials and methods” section. An *asterisks* indicates significant differences from week-matched control values as determined by 2-way ANOVA (p < 0.05) and Tukey’s post hoc test. A # indicates significant differences from week matched control values as determined by the non-parametric Kruskal–Wallis test (p < 0.05) and Dunn’s test for multiple comparisons. Values represent the mean ± SEM; n = 6 for each data point

To determine if the Cd-induced increase in urinary excretion of cystatin C was dependent on the dose of Cd, groups of rats were treated with either 0, 0.6, 1.2, or 2.4 mg/kg Cd, 5 days per week for 1–3 weeks. During the course of these studies, we found that animals receiving the highest dose of Cd (2.4 mg/kg) began to lose weight early in the treatment protocol. Accordingly, their treatment was terminated after only 1 week. Results of the complete study are shown in Fig. 4. After 3 weeks of treatment, the middle dose of Cd (1.2 mg/kg) caused significant increase in the excretion of cystatin C (graph a). The magnitude of the increase was directly related to the dose of Cd to which the animals treated. These patterns of cystatin C excretion were almost identical to those observed with Kim-1 as a marker (graph b). These results are quite similar to those observed in our previous studies on the identification of Kim-1 as an early biomarker of Cd nephrotoxicity (Prozialeck et al. 2007, 2009a, b).

**Fig. 5:**
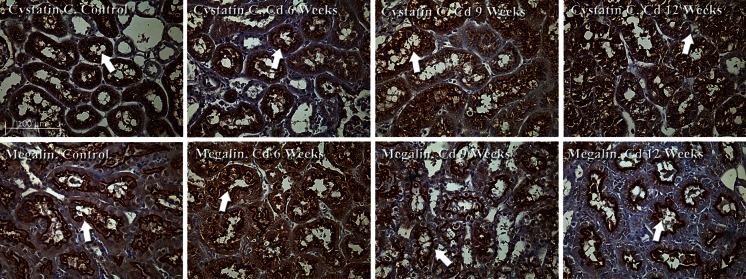
Effects of Cd on the localization of cystatin C and megalin in the proximal tubule. Animals were treated with Cd (0.6 mg/kg/day for up to 12 weeks) and formalin-fixed samples of renal cortex were processed for the immunohistochemical visualization of cystatin C and megalin as described in “Materials and methods” section. The samples were viewed using a ×40 objective. The *scale bar in the top left photo* represents 100 µm

In order to evaluate the possible functional significance of the Cd-induced increase in urinary cystatin C excretion, we utilized an immunohistochemical procedure to examine the effects of Cd on the localization of cystatin C in kidney tissue sections. As may be seen in the control sections shown in Fig. 5 (top left), fine, well-defined bands of cystatin C labeling are present just below the apical (brush border) of the proximal tubule epithelial cells (see arrow). There is also speckled, granular pattern of labeling in the cytoplasm of most cells. Cd caused pronounced changes in these patterns of labeling. In samples from Cd-treated animals, cystatin C labeling was shifted to the surface of the proximal tubule cells, making it appear as if cystatin C is actually leaking from the cells into the tubular lumen (see arrows).

In examining these images, we noticed that the general pattern of cystatin C labeling was similar, in many respects, to previously-published images showing the localization of the proximal tubular transport protein megalin (Christensen et al. 2009; Kaseda et al. 2007). Megalin is normally present on the luminal brush border surface and in the cytoplasm of proximal tubule epithelial cells where it plays a key role in mediating the uptake of low molecular weight proteins by the proximal tubule (Christensen et al. 2009; Kaseda et al. 2007; Klassen et al. 2004; Wolff et al. 2006). It has been suggested that megalin may actually mediate the uptake of cystatin C in the proximal tubule (Kaseda et al. 2007). Accordingly, we conducted a series of studies examining the effects of Cd on the localization of megalin. The results are shown in the images at the bottom of Fig. 5. As may be seen, the pattern of megalin distribution is similar, but not identical to that of cystatin C. In control samples (bottom left), megalin is mainly localized as a well-defined band of labeling along the apical surface of the proximal tubule epithelial cells although a significant amount of speckled-like labeling is present in the cytoplasm (see arrows). Interestingly Cd caused the narrowing of this band of labeling and the apparent movement of megalin from the cytoplasm to the surface of the proximal tubules (see arrows).

To quantify the apparent movement of cystatin C and megalin from the cytoplasm to the cell surface, we utilized the Image J computer program to determine the total number of peroxidase labeled pixels in cross sections of the proximal tubules shown in Fig. 5, and then calculated the percent of pixels that were located on the brush border and the tubular lumen. Results of these analyses are summarized in Table 1. As may be seen, the results clearly show that Cd caused a time-dependent shift in the localization of both cystatin C and megalin from the cytoplasm to the apical cell surface. The percentage of cystatin C associated with the apical cell surface increased from less than 15 % in the control samples to over 80 % after 12 weeks of Cd exposure.

**Table 1:** Effects of Cd on the localization of cystatin C and megalin in the proximal tubule

Sample	Total labeled pixels/tubule	Total labeled pixels on apical surface	Percent of labeled pixels on apical surface
Cystatin C			
Control	94,767 ± 9490	12,515 ± 3026	12.1 ± 2.1
Cd 6 weeks	72,159 ± 5954	26,355 ± 3960	35.7 ± 4.1*
Cd 9 weeks	127,880 ± 27,992	107,114 ± 23,663	76.1 ± 2.2*
Cd 12 weeks	153,179 ± 12,307	136,144 ± 10,923	89.0 ± 2.0*
Megalin			
Control	166,802 ± 25,904	45,788 ± 6808	30.3 ± 3.1
Cd 6 weeks	115,589 ± 15,109	48,167 ± 6565	41.2 ± 1.0*
Cd 9 weeks	98,532 ± 13,923	51,438 ± 6580	52.9 ± 1.8*
Cd 12 weeks	124,177 ± 9758	83,655 ± 7.384	67.1 ± 1.8*

The localization of cystatin C and megalin in the peroxidase-labeled kidney sections from Fig. 5 were quantified using the Image J computer program. Using the hematoxylin staining as a morphologic guide, regions of interest were created around each tubule. The number of pixels that were positive for cystatin C or megalin staining was measured using constant thresholding tools (as described in “Materials and methods” section) to identify peroxidase signal above background levels for each tubule. A second region of interest (apical compartment) for each tubule was created to encompass the luminal space and 1–2 cells of the apical luminal surface. Positive cystatin C and megalin pixels were again measured in the apical compartment using the same thresholding parameters. The percentile data of pixels in the apical compartment was calculated and the data were evaluated by a one-way ANOVA with post hoc Tukey’s test. Values represent the mean ± SEM of data from 7 to 10 tubules per image. An *asterisks* denotes that the percent of pixels in the apical compartment is significantly greater (p < 0.05) than in the control samples

One interesting aspect of the data in Table 1 is that the total cystatin C labeled pixels in the proximal tubules appears to increase with Cd treatment. This is somewhat surprising in that Cd is actually increasing the urinary excretion of cystatin C. Intuitively, it would seem that cellular levels of cystatin C should actually decrease. While we cannot explain this apparent paradox, we are postulating that it may reflect inhibitory effects of Cd on the catabolism of cystatin C within the proximal tubule cells, although further studies are needed to clarify this issue. It is also noteworthy that the pixel intensities for megalin show that Cd had no consistent effects on the overall intensity of megalin labeling, even though it did increase the percentage of megalin on the apical cell surface.

**Fig. 6:**
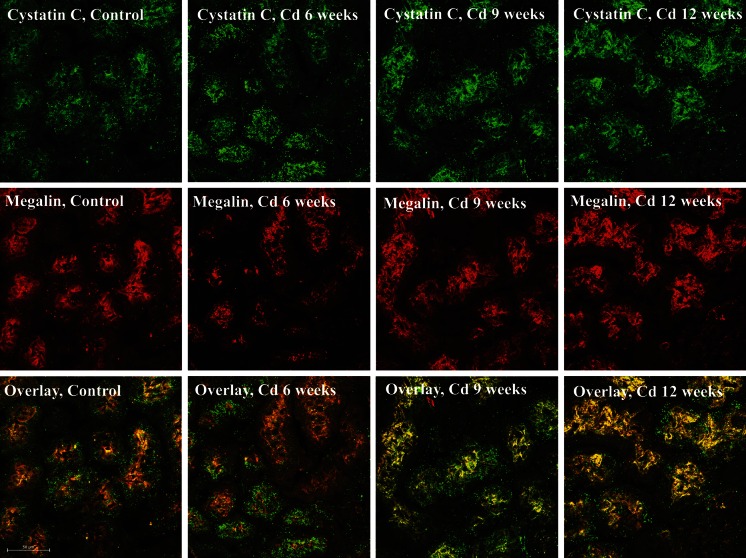
Effects of Cd on dual labeling of cystatin C and megalin in renal cortex. Animals were treated with Cd (0.6 mg/kg) for 6, 9 or 12 weeks. Cryosections of renal cortex were processed for the dual labeling and confocal imaging of cystatin C and megalin as described in “Materials and methods” section. In these images, cystatin C (*top row of images*) shows as green fluorescence, whereas megalin (*middle row of images*) is visible as red fluorescence. The *bottom row of images* show overlays of the cystatin C and megalin images from the same tissue sections. The samples were viewed using a ×60 objective. The *scale bar on the bottom left image* represents 50 µm

To further examine the possible association between the effects of Cd on cystatin C and megalin, we developed a dual immunofluorescence and confocal procedure for the visualization of both molecules in the same tissue cryosections. As may be seen in Fig. 6, the patterns of labeling for both cystatin C and megalin with this dual fluorescence method are similar to those observed with the individual immunoperoxidase labeling studies described in Fig. 5. In control samples, cystatin C showed a fine band of labeling just below the apical cell surface and a speckled, granular pattern of labeling in the cytoplasm, whereas megalin showed a well- defined band of labeling along the apical cell surface as well as speckled labeling in the cytoplasm. It should be noted that overlaid images of both molecules together showed that even though the two molecules showed similar patterns of labeling, they were not exactly co-localized. This is evident in the control samples that show mainly distinct regions of red megalin labeling and green cystatin C labeling with only isolated areas of yellow labeling where both molecules appear to be co-localized. By contrast, in the Cd treated samples, there appeared to be a movement of both molecules toward the apical cell surface. Overlaid images of both molecules showed Cd-dependent increase in the appearance of yellow labeling indicating an increase in the co-localization of both molecules as they relocalized to the cell surface and the tubular lumen.

In order to quantify the apparent Cd-induced increase in the co-localization of cystatin C and megalin, we utilized the Image J program to examine the correlation between the locations of the molecules. After background subtraction, the localization of megalin and cystatin C in the control samples had a Pearson’s correlation coefficient of 0.54 and a Manders co-localization coefficient of 0.87 (correlation of megalin pixels that reside in the same location as cystatin C pixels). Over time, however, Cd exposure altered the localization patterns of cystatin C and it co-localized more strongly with megalin. For 9 weeks and 12 weeks of Cd exposure, the Pearson’s correlation coefficients between cystatin C and megalin were 0.70 and 0.78, and the Manders co-localization coefficients were 0.95 and 0.91, respectively. It should be noted that these particular images could not be used for more sophisticated statistical analyses because the fluorescence data in each image is essentially treated as n = 1. This is in contrast to the immunoperoxidase images shown in Fig. 5 in which each tubule can be treated as n = 1. Nevertheless, the results of the fluorescence analyses clearly indicate an increase in the co-localization of cystatin C and megalin in the samples from the Cd-treated animals. In addition it is worth noting that the total pixel intensity for the fluorescent megalin images did not appear to change with Cd exposure, whereas the intensity of the cystatin C labeling increased steadily over the 12 week Cd treatment protocol. Even though no statistical analyses could be performed on this data, the results are in agreement with the quantitative immunoperoxidase data summarized in Table 1.

## Discussion

Cystatin C is an emerging marker of both ischemic and nephrotoxic kidney injury (Ghys et al. 2014). Serum levels of cystatin C have been proposed as an alternative to traditional markers such as BUN and creatinine for the estimation of glomerular filtration rate (Ghys et al. 2014). At the same time, increases in the urinary excretion of cystatin C have been proposed as a sensitive indicator of proximal tubular injury (Ghys et al. 2014; Togashi et al. 2013; Wallin et al. 2014). The fact that cystatin C is rapidly being accepted as a measure of renal function in the clinic makes it extremely important to understand the effects that specific nephrotoxic agents, such as Cd, have on serum and urinary levels of cystatin C. While several recent studies have addressed the possible utility of cystatin C, a potential marker of Cd nephrotoxicity in Cd-exposed human subjects, the studies have primarily focused on measurement of serum levels of cystatin C and have yielded conflicting results (Harisa et al. 2014; Kim et al. 2012; Poreba et al. 2011; Shelley et al. 2012; Wallin et al. 2014; Weaver et al. 2011; Wu et al. 2011). The results of the present studies utilizing a well-established model of sub-chronic Cd exposure in rats indicate that an increase in the urinary excretion of cystatin C is a very early and sensitive marker of Cd-induced proximal tubular injury. Significantly elevated levels of cystatin C appeared in the urine after 3–6 weeks of treatment with Cd, whereas overt polyuria and proteinuria, which are classic signs of Cd-induced proximal tubule injury, did not become evident until 9–12 weeks of Cd treatment. Dose–response studies showed that the magnitude of the increase in the urinary excretion of cystatin C was directly related to the dose of Cd to which the animals were exposed.

In order to determine whether the effects of Cd on cystatin C may have involved alterations in glomerular function, serum levels of cystatin C were measured. As noted previously, cystatin C in the serum is normally filtered at the glomerulus and then taken up by the proximal tubule. In theory, alterations in glomerular filtration of cystatin C could lead to changes in serum levels of cystatin C. It should be noted that the serum levels of cystatin C reported here are consistent with other studies reported in the literature (Dieterle et al. 2010; Ghys et al. 2014; Togashi et al. 2013). We found that Cd had no effect on serum cystatin C, which indicated that cystatin C was being filtered normally by the kidney, and that the increase in urinary cystatin C was most likely due to a reduction in the reabsorptive capacity or alterations in the catabolism of cystatin C in the proximal tubule. Furthermore, H&E stained tissue sections (Fig. 3) showed no evidence of changes in the structure of the glomeruli. Together, these results strongly indicate that the primary site of Cd-induced kidney injury in this model is in the proximal tubule.

In assessing the utility of various biomarkers of Cd nephrotoxicity such as cystatin C, β_2_ microglobulin and Kim-1, it is important to note that each of these molecules is derived from different sources and their appearance in the urine is indicative of different events in the pathophysiology of kidney injury (Prozialeck and Edwards 2010, 2012; Thevenod and Lee 2015). The classic view is that with chronic exposure, the levels of Cd in the proximal tubule cells continue to accumulate until a critical threshold concentration of about 150–200 µg/g of tissue is reached (Jarup 2002; Roels et al. 1979). At this point, cell function becomes compromised and the injury can trigger autophagy and either necrotic or apoptotic cell death (Prozialeck and Edwards 2012; Shaikh et al. 1999; Thevenod and Lee 2015). Along with causing alterations in proximal tubule function, cell injury leads to the shedding of injured cells and their cytosolic contents into the urine. The shedding of dead or injured cells triggers a repair process in which neighboring non-injured cells dedifferentiate, migrate to the denuded area of the basement membrane and proliferate to replace the injured cells (Prozialeck and Edwards 2012; Thevenod and Lee 2015).

With respect to biomarkers, Kim-1 is mainly expressed as part of the tissue repair process when injured cells slough off from the proximal tubule and surviving cells migrate to the denuded areas of the basement membrane to reform the epithelial barrier (Bailly et al. 2002; Ichimura et al. 2008). By contrast, increases in the urinary excretion of classic low molecular weight protein markers derived from serum such as β_2_ microglobulin and cystatin C are thought to directly reflect the reabsorptive dysfunction that results from Cd-induced injury to the proximal tubular epithelial cells. In light of the fact that the proximal tubule transport protein megalin has been implicated in these reabsorptive processes (Christensen et al. 2009; Kaseda et al. 2007; Klassen et al. 2004; Wolff et al. 2006), we examined the possible relationship between changes in the localization of cystatin C and megalin in the proximal tubule. Results of direct labeling studies clearly showed that Cd caused the relocalization of megalin and cystatin C from the cytoplasm to the apical surface of the epithelial cells of the proximal tubule. Interestingly, preliminary analysis of pixel intensity for megalin labeling showed no difference between tissue samples from control and Cd-treated animals even though in vitro studies have suggested that Cd can reduce transcription levels of megalin (Gena et al. 2010). At present, the mechanism by which Cd affects the localization of cystatin C in the proximal tubule remains unclear. However, the present findings do suggest an association with alterations in megalin distribution and/or function.

It should be noted that while the present discussion focusses on the possible role of megalin in the uptake of cystatin C, there is evidence to suggest that megalin is also involved in the uptake of other Cd biomarkers such as β_2_-microglobulin and metallothionein. This raises the intriguing possibility that Cd might affect the renal excretion of all of these markers by disrupting megalin-mediated transport. Results of the present studies showing that the time courses for the urinary appearance of cystatin C and β_2_-microglobulin are very similar indicate that they may in fact involve a common mechanism. However, previous studies from our laboratory (Prozialeck et al. 2007, 2009b) showed that metallothionein, which is another substrate for megalin transport (Klassen et al. 2004), typically does not appear in the urine until about 6–9 weeks of exposure, about 3 weeks after cystatin C and β_2_-microglobulin. We are postulating that such differences in the time course for the appearance of megalin substrates in the urine could be related to differences in the actual levels of the marker molecules, the kinetics of their transport by megalin and/or their handling by the epithelial cells of the proximal tubule. It is noteworthy that Thevenod and Wolff (2015) have recently presented evidence that metallothionein is not endocytosed in the proximal tubule via megalin because the concentration of metallothionein in the primary filtrate is too low to be able to bind megalin. These authors propose that metallothionein may rather be endocytosed by a high- affinity receptor in the distal nephron. Our previous work demonstrating that metallothionein is detected in the urine several weeks after cystatin C and β_2_-microglobulin (Prozialeck et al. 2007) could suggest that this delay of urinary metallothionein excretion could either reflect altered reabsorption of metallothionein in the distal nephron or the release of cytosolic metallothionein from damaged proximal tubule cells into the urine. Further studies are needed to clarify these issues and to determine how the findings by Thevenod and Wolff relate to our findings on the urinary excretion of cystatin C.

In addition, there are several other caveats that need be considered. First, the present studies merely show an association between changes in cystatin C and megalin localization. They do not prove any cause-effect relationships. Secondly, at this time we can only speculate about alternative mechanisms by which Cd could affect the uptake of substrates such as cystatin C. For example, Herak-Kramberger et al. (1998) have shown that Cd blocks endocytes in proximal tubule cells by inhibiting endosomal V-ATPase activity. It is also possible that some of the changes in the localization of molecules such as megalin and cystatin may occur secondarily to the disruption of cell–cell adhesion and cytoskeletal disruptions that occur during the early stages of Cd-induced proximal tubule injury (for review see Prozialeck and Edwards 2012).

The results of the present studies show that the time course for the Cd-induced increase in urinary cystatin C excretion occurs at about the same time as the increase in urinary β_2_ microglobulin excretion. Since the increase in the excretion of both proteins probably results from alterations in proximal tubular reabsorptive function, the obvious question that arises is, does one marker offer any advantages over the other? In this regard it is worth noting that while β_2_ microglobulin is an early marker of proximal tubule damage, urine levels can also be altered by pathology and age-related changes in organs other than the kidney (Bokenkamp et al. 2001; Prozialeck and Edwards 2010). Cystatin C, on the other hand, is produced by tissues across the body at a stable rate regardless of tissue damage and/or metabolic state (Filler et al. 2005; Sharma et al. 2009) which potentially makes cystatin C a more reliable measure of proximal tubule function than β_2_-microglobulin.

Another potential benefit of cystatin C as a biomarker for Cd-induced renal injury is that it is conserved across species and is an indicator of renal injury in a variety of species (Ghys et al. 2014). The availability of another standard marker that is covered across species could greatly facilitate the extrapolation of the results of studies in animals to the results of studies on human populations and vice versa.

One important point to keep in mind with all of the biomarkers discussed in this report (cystatin C, β_2_ microglobulin and Kim-1) is that they are very sensitive, but also general, markers of kidney injury. Even though they are sensitive markers of injury, none of the markers are specific indicators of Cd toxicity *per se.* Thus, even with the use of these biomarkers to detect kidney injury, appropriate patient histories and direct analyses of Cd in tissues and fluids are still necessary for the appropriate diagnosis of Cd-induced kidney injury (Prozialeck and Edwards 2010, 2012). However, the availability of sensitive markers such as cystatin C may allow for more effective screening and, perhaps more effective treatment options and clinical outcomes.

A final aspect of this study that merits attention involves the relevance of the animal model to the typical patterns of Cd exposure in humans. All of the present studies involved the subcutaneous administration of Cd at doses ranging from 0.6 to 2.4 mg/kg body weight for up to 12 weeks. This is a standard protocol that has been widely used by a variety of investigators in the Cd field including our own initial studies on the use of Kim-1 as a marker of Cd toxicity (Prozialeck et al. 2007, 2009a, b; Prozialeck and Edwards 2012). We would like to emphasize that even though it might appear that administering Cd by the subcutaneous route would not mimic the manner in which humans are typically exposed to Cd (chronic oral ingestion or inhalation), it actually does. Moreover, this protocol offers many advantages for these types of studies. In utilizing in vivo models to study Cd nephrotoxicity, investigators must balance the need to be able to do the studies in a reasonably short time frame with the need to replicate the toxicokinetics of the long-term, low-level patterns of exposure that are common in humans who are typically exposed to dietary or inhaled Cd over many years or decades. It simply is not possible or practical to replicate these types of exposure in species such as rats or mice. Since these species have shorter life spans, and for many practical reasons, exposure levels used in animal studies must be higher, but shorter in duration, than those of humans.

Cd is a classic cumulative nephrotoxicant. With higher levels of exposure, nephrotoxic effects occur more quickly than with lower levels of exposure. There is a direct inverse linear relationship between the dose of Cd and the time of exposure that causes onset of proximal tubule injury (i.e. doubling the dose produces effects in ½ the time) (Prozialeck et al. 2007). However, higher doses of Cd can cause injury to organs other than the kidney, particularly the liver and gonads. One of the most useful approaches for nephrotoxicity research has involved the subcutaneous administration of moderate doses of Cd (0.3–1.2 mg/kg/day) for periods ranging from 3 to 12 weeks. With this protocol, it is possible to accurately control the dosing of the animals and to produce the full spectrum of Cd’s nephrotoxic effects, ranging from mild to severe. In addition, the patterns of Cd distribution and toxicity with this model are comparable to those with chronic oral exposure (for review see Prozialeck and Edwards 2010). Most importantly, since this protocol has been used extensively and is widely accepted as a standard in the Cd field (Aoyagi et al. 2003; Dudley et al. 1985; Goyer et al. 1989; Shaikh et al. 1999) it allows for the comparison and interpretation of results across different studies.
